# Psychological Analysis of Religiosity and Spirituality: Construction of the Scale of Abandonment by God (SAG)

**DOI:** 10.1007/s10943-021-01197-7

**Published:** 2021-03-06

**Authors:** Stanisław Głaz

**Affiliations:** Department of Philosophy, Institute of Psychology, Jesuit University Ignatianum, Ul. Kopernika 26, 31-501 Kraków, Poland

**Keywords:** Religiosity, Spirituality, Abandonment by God, Scale of abandonment by god (SAG)

## Abstract

The issue of religiosity and spirituality and their measurement are quite well developed fields in the psychology of religion. However, the literature shows a lack of research tools to measure the religious experience of the feeling of abandonment by God among followers of the Catholic religion. The purpose of this article is to fulfill this gap through the presentation of the notion of ‘God abandonment’, and its operationalization, by constructing the Scale of Abandonment by God: SAG (Skala Opuszczenia Przez Boga—SOPB). The psychometric value of the tool was evaluated, that is the reliability and validity. In order to achieve this goal, three stages of instrument development (item generation, scale development, and instrument testing) were undertaken in three studies. Stage 1: The pilot study concerned the development of positive statements about the concept of the Catholic experience of God (i.e., the subjective feeling of the experience of God's abandonment in the life of a contemporary person, as well as showing to what extent this belief can affect some aspects of his/her life). Stage 2: Was designed to perform exploratory factor analysis and test–retest reliability to assess stability of the SAG in a three-week time range. Stage 3: Validation of the SAG by Confirmatory Factor Analysis was performed. Result: The SAG can be recognized as a one-factor measure of the feeling of abandonment by God. Because the content of the SAG items indicate the positive aspects of the abandonment of God, this can assist people living in Catholic societies.

## Introduction

Many researchers have developed definitions of “religiosity” and “spirituality” (Jaworski 1989; Hill and Hood 1999; Oman 2013). Due to their complex structure and cultural conditions (Vetter 1991; Abu-Raiya and Pargament 2015), it is assumed that religiosity and spirituality (RS) can be understand as dimensions of human experience that involve beliefs, practices, and experiences related to a transcendent, sacred reality (Huber 2003; Głaz 2013a). Although interrelated RS are empirically and theoretically distinct, religiosity involves behaviors related to organized traditions, whereas spirituality usually refers to personal beliefs and experiences (Miller and Thoresen 2003). According to some researchers (Szymołon 2011; Park et al. 2017), the conceptual model of RS comprises many specific components. They collectively represent a constellation of important affective, behavioral, and cognitive variables. RS can play an important and positive role in human lives (Pargament 1997), provided that these are mature (Allport and Ross 1967) and personal (Jaworski 1989). RS mainly perform cognitive functions. Man derives knowledge from various sources and during personal contact with God (Głaz 1998; Huber 2003). RS sometimes play an important role as a prophylactic and preventive factors against disorders and pathologies in human life (Zavalloni 1971). These can often be an important buffer factor in the face of disorders and dysfunctions of an individual, playing also a social rehabilitation role (Jaworski 1989; Wnuk and Marcinkowski 2012). Moreover, authentic, internal RS play an important role in the development of human potential and abilities (Oman 2013), and a significant role in the process of self-realization (Vries-Schot et al. 2008). The result of this process is an authentic, mature personality (Jaworski 1989), which manifests many creative features such as reflexivity, independent thinking, openness, independence (Allport and Ross 1967; Pargament 1997). People who define themselves as RS and who identify themselves with a specific religious tradition tend to be less depressed and have greater self-esteem (Krok 2015), more effective coping skills (Pargament et al. 2001), greater happiness and greater life satisfaction (Gautherier et al. 2006), and better physical health (Schnittker 2001). Behaviors such as reliance on God, praying, and going on pilgrimages can promote comfort and mental health through peace and develop hope and positive thinking. RS beliefs increase resistance against disasters and thus help preserve physical and mental health, prevent infliction of diseases, and finally promote hopefulness (Khodapanahi and Khaksar 2005; Cohen et al. 2009). Gorsuch and Hao (1993) found that higher religiosity of individuals was associated with more self-reported motivation both to forgive and to work harder to be forgiven. Research by Jaworski (2006) showed that there is a positive relationship between prayer and happiness, faith and independence, religious self and the value of salvation. Orthodoxy dimension of religiosity correlates with suspiciousness and guilt proneness (Śliwak and Zarzycka 2012). Głaz’s (2013a, 2014a, 2014b) research conducted among students showed that there is a positive relationship between the experience of God and social values, and the sensitivity of conscience, and the sense of meaning in life. The self-concept and quest religious orientation were found to be a predictor of self-esteem, which indicates a mediating effect of this religious orientation (Błażek and Besta 2012).

Researchers have developed a number of RS measurement tools, I mention only some of these. The early used methods were qualitative that focussed open-ended responses in religious studies related to RS. For example, David and Sally Elkind (1973) asked adolescents to answer the questions such as: “When do you feel closest to God…?”. Such methods used in the description of RS demonstrated to be insufficient and incomplete.

Therefore, researchers tried to develop religious quantitative measures. It is worth to pay attention to some of these. The following inventory is based on a review of the literature in the field, both from a psychological and a theological perspective. Researchers constructed quantitative tools to study the RS in relation to the Christian religion (Hill and Hood 1999; Huber 2003) and for other religious faiths. For example: “Brief Orthodox Jewish Religiosity Scale” (Pirutinsky 2009), “Muslim Daily Religiosity Assessment Scale (MUDRAS)” (Olufadi 2016). Others developed tools to assess attitudes toward religion, such as "Francis scale of Attitude toward Christianity” (FSAC) (Francis and Stubbs 1987). Joseph and DiDuca (2007) developed “Dimensions of Religiosity Scale” to assess four dimensions of religious behavior and thinking: (1) preoccupation, (2) guidance, (3) conviction, and (4) emotional involvement. Vries-Schot de Pieper and Uden van (2012) developed a tool for measuring Christian religiosity “The Mature Religiosity Scale.” Paloutzian and Ellison (1982) developed a two-dimensional scale for measuring spiritual well-being “The Spiritual Well-Being Scale.” It distinguishes between two related but separate aspects: religious well-being and existential well-being. For the measurement of the affective relationship between man and God, Edwards (1976) developed “The Religious Experience Questionnaire.” In recent years, Huber (2012), based on the theoretical model of religiousness of Stark and Glock (1968), which is a multidimensional construct, developed a research tool for measuring Christian religiosity, “The Centrality of Religiosity Scale.” Exline et al. (2014) constructed a tool to measure religiousness and spirituality including comfort and tension “The Religious and Spiritual Struggles (RSS) Scale.” Głaz (2020) has designed “The Intensity of Religious Experience Scale (IRES)” that deals with experiencing God’s presence and God’s absence in human life.

## Genesis of the Scale of Abandonment by God (SAG)

Following the methodology of constructing the research tool, theoretical understanding of the Catholic RS as the basis of the current scale is indicated. It is related to Catholic anthropology and the Catholic concept of God (Głaz 1998). The Catholic concept of God presents God with personal characteristics as the God who revealed himself in Jesus Christ. Moreover, in proper time, God sent out the Holy Spirit. The Holy Trinity participates in the sanctification and salvation of man (Rahner 1984). On the other hand, the Catholic anthropology suggests that man realizes himself as a person, while also realizing his talents, predispositions, and abilities in relation to others and to God (Vergote 1967). Man has a complex structure, which consists of the biological, physiological, mental and spiritual dimensions (Rahner 1984; Frankl 1987) that create a whole. All components of the structure interact with each other, performing specific functions. The mental dimension is related to the mental life of a person (Frankl 1987; Huber 2003). The spiritual dimension being exclusive human property. It directs people toward one another, and toward God (Głaz 2013a) and allows a person to get to know, reflect, make decisions (Grom 2007).

John of the Cross explains that God can live in a threefold way in the soul of man (St. John of the Cross 2007, 11, 1–3). The first way is through His being the One who gives life and sustains it. The second type of presence is through grace. God communicates Himself to man in His own way. The third type of presence is through spiritual feeling. Man is aware that God is present in his life, and he feels His presence in himself. He refreshes and enriches human’s life. Often this kind of experience is connected with the experience of God's abandonment. The abandonment by God does not mean that God has left the world, and the human, although the man may not feel His presence in own life. The man has a feeling that God has abandoned him, that God has departed from him, not necessarily because of his own sins or neglect, but then, for unknown reasons, after some time God will return to him again. The experience of being abandoned by God is a common experience of a religious person (Rahner 1984; Grom 2007). It is often associated with the normal process of RS development in a human being (Jung 2010). At times, it can be the greatest test of faith imaginable for a person (Głaz 1998). This kind of experience can contribute to the emergence of such feelings as anxiety, confusion, and loneliness. It leaves a trace on human life. The understanding of abandonment by God can be also analyzed within the religious meaning system (e.g., Krok 2014; Krok et al. 2019) as it bears relevance from a cognitive point of view. The religious meaning system reflects the fact that religion enables individuals to explain and interpret reality in the categories of significance and purpose. The feeling of being abandoned by God may thus be a consequence of previous cognitive reinterpretation of the individual’s beliefs. Literature and research reveal that it also brings something good to human life (Głaz 2013b). It plays a positive role. It awakens a longing for God, provides new religious content, knowledge about oneself and others, and sometimes it encourages deepening one's faith toward God and love toward others, and stimulates people to become more involved in religious life (Głaz 1998, 2013b). The effects of the experience of being abandoned by God are visible in the religious and personality sphere of a human related to his personal and social life (Meister Eckhart 1955; St. John of the Cross 2007; Głaz 2013b) and is integrated into the overall human experience (Jung 2010). It involves the cognitive, emotional, and behavioral dimensions of a person. It can sometimes be culturally and socially conditioned by religious differences (Głaz 1998; Huber 2003). The aforementioned Catholic theoretical conception of the RS will serve to construct a tool for measuring the experience of God's abandonment.

## Construction of the SAG

There are several reasons why an attempt was made to construct the SAG scale. One wants to know to what extent the proposed theory of Catholic experience of the abandonment by God is scientific, true, and verifiable. As it was mentioned above, in recent years, many standardized tools for RS examination have been developed (Jaworski 1989; Hill and Hood 1999; Szymołon 2011). In Poland, foreign-language research tools were also culturally adapted to local conditions (Zarzycka 2007, 2014). However, there is still a lack of standardized RS measurement tools connected to people living in the Catholic religion environment. The tools for measuring RS so far seem insufficient, and the rich structure of RS requires empirical verification of theoretical studies. This is especially true of the specificity of the Catholic experience and also the lack of this type of tool. There are numerous countries where many Catholics live. For example Poland, where over 90% of Polish citizens declare membership of the Roman Catholic Church (the Institute of Statistics of the Catholic Church 2019). Many people experience frequent interaction with the transcendent as a fundamental part of life. It can be also seen the departure of believers from the institutional religiosity toward internal religiosity. New religious groups declare that they attach great importance to the subjective dimension of RS. Moreover, it is noted that this type of religious experience takes place in the life of contemporary man. Despite the fact that many researchers show its negative influence on the development of religious life and personality of an individual, there are only few researchers who perceive its positive influence on development of an individual's religiousness and personality (Cohen et al. 2009; Mariański 2011; Szyszka 2018).

Therefore, the following hypotheses are stated.

### H_1_

It exists experience of God's abandonment in the life of contemporary Catholic man measured by SAG.

### H_2_

The SAG scale has the sufficient psychometric properties.

## Validation of the SAG

The description of the construction and validation of the SAG was done according to the following procedure. The study was conducted in three study steps. Study 1 was to get statements related to the concept of Catholic experience of abandonment by God, and then evaluating them, and generating them for further analysis (pilot study). Study 2 was carried out to perform the exploratory factor analysis and to assess the three-week test–retest reliability of the SAG. Study 3 was carried out to perform the confirmatory factor analysis.

### Study 1: Pilot Study

In order to test the stated hypotheses, the three studies were conducted. The first, the pilot study was aimed to check the generated principal statements, the items of the SAG, whether they are understandable and correctly understood, and to verify if they actually measure what was intended to be measured, i.e., to check if they mirror the adapted theoretical concept of the Catholic religious experience of abandonment by God.

To collect the proper material served to develop the initial pool of items for SAG involving understanding positive aspects of Catholic experience of abandonment by God the surveys were conducted during interviews in pastoral work, and taken from the relevant scientific literature. Primarily, the statements were evaluated by a group of students in scientific seminars at an university. The statements, which content was similar among themselves and incomprehensible, were dropped out. The generated list of face-validated 14 statements was accepted for further analysis. Next, the group of experts, theologians, and psychologists assessed the pool of 14 items. They paid attention to the theoretical correctness of each statement, according to the method proposed by Lawshe (1975), on a three-point scale. Fourteen statements were evaluated due to the following scale: (3 = *this statement is essential for a given scale*, 2 = *this statement is useful, but it is not essential for a given scale*, 1 = *this statement should not be in a given scale*). According to the experts, useless or ambiguous 5 statements were kicked out. Nine statements were accepted for further analysis.

### Study 2: Assessment of SAG Statistical Properties

In order to determine the statistical properties of SAG, the empirical study was conducted. Each item was evaluated on the 7 point scale, 7 = *definitely yes* (I *definitely agree*), 6 = *yes* (*I agree*), 5 = *rather yes* (*I rather agree*), 4 = *I cannot decide*, 3 = *rather not* (*I rather don't agree*), 2 = *not* (*I do not agree*), 1 = definitely not (*I definitely disagree*). The obtained data were used to verify the scale validity. The exploratory factor analysis and three-week test–retest reliability were used. The factor analysis revealed one-factor solution, what was in accordance with the expected Catholic theory of RS and the adapted assumptions.

## Method

### Participants and Procedure

A total of 392 undergraduate full-time and part-time students from a public university in Kraków took part in the study. The age of the respondents ranged from 19 to 80 (*M* = 54.2; *SD* = 13.32). 47.3% of the surveyed population were men and 52.7% women. Students were asked about their place of birth, belonging to a religious group, as well as religious commitment. All persons grew up in a Catholic family and were born in Poland, who consider themselves as believers and practitioners. They take part in the Sunday Eucharist, they pray and take sacraments. They had in their lives the experience God’s abandonment. The response for the question about the frequency of the experience of abandonment by God is as follow: 14.5% of the respondents stated that they had this experience very often, 48% frequently, 26% rarely, and 11.5% very rarely. In order to receive material for the further construction of the scale, people were asked to respond to each item. Each participant responded to the all statements on a 7-point Likert-type scale, about the extent in which he or she agrees or not with the statement. The people who did not respond to all test items were excluded from the analysis. There were 21 of them. The results obtained on the basis of 371 correctly completed questionnaires were used for analysis.

## Results

### Exploratory Factor Analysis of the SAG

The received data, obtained in the sample, were subject to exploratory factor analysis. To determine the validity, the results were analyzed using the Varimax rotation. Matrix of factor loadings is presented in Table 1.

**Table 1:** Factors loadings of the SAG items (*N* = 371)

Items	The content of items regarding the Scale of Abandonment by God	*F*1
7	Although I am accompanied by a temporary feeling of abandonment by God, my life seems meaningful and valuable. *Mimo że towarzyszy mi chwilowe poczucie opuszczenia przez Boga moje życie wydaje się sensowne i wartościowe*	.872
2	Even though I am accompanied by a feeling of abandonment by God, I still strive to deepen my relationship with Him. *Mimo że towarzyszy mi poczucie opuszczenia przez Boga, ciągle zabiegam o pogłębienie relacji z Nim*	.778
4	After experiencing the feeling of God's departure, I have a feeling sense of longing for Him. *Po doświadczeniu poczucia odejścia Boga towarzyszy mi uczucie tęsknoty za Nim*	.710
1	Even though I have a feeling of being abandoned by God, I am convinced that God loves me. *Nawet jeśli mam poczucie opuszczenia przez Boga, jestem przekonany, że Bóg mnie kocha*	.706
3	Despite the feeling of being abandoned by God, I still hope to experience His presence again. *Mimo poczucia opuszczenia przez Boga, ciągle mam nadzieję, że doświadczę jeszcze jego obecności*	.689
5	Despite the feeling of being abandoned by God, I do not think that God could abandon me because of my sins and my neglect. *Mimo poczucia opuszczenia przez Boga nie uważam, że Bóg mógłby mnie opuścić z powodu moich grzechów i zaniedbań*	.633
6	Even though I have a feeling of being abandoned by God, I never question that God is present in my life. *Nawet jeśli mam poczucie opuszczenia przez Boga, nigdy nie kwestionuję, że Bóg jest obecny w moim życiu*	.622

For further analysis, all items (statements) with loadings greater than 0.6 were taken into account. (Table 1). Two statements with factor loadings less than 0.6 were excluded from analysis. “The feeling of being abandoned by God does not help you get to know yourself better” (0.532) and “The feeling of being abandoned by God helps to deepen religious faith” (0.521). The SAG explains 47.8% of the variance of the abandonment by God. The factor loadings of the items are strong. The obtained statements correlate positively with each other (Table 2). The obtained correlation coefficients between the statements are from *r* = 0.27 to *r* = 0.63. This suggests that the accepted statements of the scale related to Catholic experience of the abandonment by God confirm the theoretical structure this experience and form the basis for further analyzes.

**Table 2:** Correlation coefficients *r*-Pearson’s for each statements (*N* = 371)

Items	2	4	1	3	5	6
7	.63**	.58**	.56**	.51**	.47**	.50**
2	–	.54**	.43**	.46**	.36**	.38**
4	–	–	.37**	.45**	.30**	.27*
1	–	–	–	.37**	.38**	.39**
3	–	–	–	–	.32**	.29*
5	–	–	–	–	–	.37**

## Reliability and Stability Analysis of the SAG

The reliability of the scale was estimated by calculating Cronbach's alpha. Table 3 presents descriptive statistics and reliability coefficients of SAG. Stability was measured using three-week test–retest method.

**Table 3:** Descriptive statistics and reliability indicators for the SAG (*N* = 371)

SAG	*M*	*SD*	Minimum	Maximum	Alfa Cronbacha
	5.5	.939	2.00	7.00	.89

The reliability index of the SAG is high (Table 3). Cronbach's value (0.89) for scale is satisfactory, which displays a high reliability of the measure. The result obtained on the scale is high (*M* = 5.5), and standard deviation (*SD*) is 0.939.

The stability of the SAG was estimated by a test–retest method with an interval of three weeks. The average results obtained among 58 people from the test (*M* = 5.4; *SD* = 0.982) and retest (*M* = 5.2; *SD* = 1.09) are very similar. The correlation between the results from the first and second studies affirmed the stability of the scale. The correlation coefficient is *r* = 0.82, *p* < 0.001. The SAG has a high stability indicator.

### Study 3: Confirmatory Factor Analysis and Validation of the SAG

In order to examine the hypothesis 2, theoretical validity of the scale was checked through the use of confirmatory factor analysis and criterion validity of the scale by calculated correlations with other known scales in the field.

## Method

### Participants and Procedure

The research was conducted among the inhabitants of the Krakow region. Detailed instructions on how to complete the questionnaire were provided for the participants. People were asked about their place of birth, belonging to a religious group, as well as religious commitment. The demographic information obtained from the respondents includes age and gender. All respondents were born in Poland and grew up in a Catholic family. All the people surveyed declared that they were believers and practitioners. They take part in the Sunday Eucharist, they pray and take sacraments. They had in their lives the experience of God’s abandonment. In order to obtain material for the confirmation of the obtained model, people were asked to respond to each SAG statement as well as to the two additional questionnaires. The analysis excluded those of people who did not respond to all test items. There were 11 of them. The results obtained from 200 correctly completed sets of questionnaires of people were used. The age of the respondents ranged from 19 to 68 (*M* = 41.12; *SD* = 13.23). Most of the participants in this confirmatory study were women. 41.8% of the surveyed population were men and 58.2% women.

## Measures

- Participants of the third study completed the final SAG version on the 7-point scale as developed and described in previous Study 2. The Cronbach's alpha coefficients for the current study were 0.90 for the SAG scale. The results obtained in the scale are high (*M* = 5.4), standard deviation (*SD*) is 0.931. In addition, participants were asked to complete the following questionnaire.
- The Centrality of Religiosity Scale (CRS) by Huber was used to measure Christian religiosity (Huber and Huber 2012). The task of the examined person is to answer each question by choosing the appropriate answer spread on the 5 point scale: 1. *not at all* 2, *slightly*, 3. *on average*, 4. *rather*, 5 *very*. The CRS scale defines five dimensions of Christian religiosity: interest in religious issues, the intellectual dimension (ZPR); religious beliefs, dimension of ideology (PR); prayer, dimension of private practice (M); religious experience, the dimension of religious experience (DR) and worship, dimension of public practice (K). Zarzycka (2007) adapted it to Polish conditions. The reliability of the scale was estimated using Cronbach's alpha: 0.82 ≤ α ≤ 0.90. The obtained measures of correlations between the results obtained in the first and in the second studies confirmed the stability of the scale (*r* = 0.62 – *r* = 0.85). In the current study, Cronbach's alpha coefficient is: 0.81 ≤ α ≤ 0.89.
- Religious Comfort and Strain Scale (RCSS) (Exline et al. 2000, 2014). It is a set of 24 face-valid items designed to assess the degree to which participants are experiencing feelings of comfort and three types of struggle associated with Christian religiosity. Participants are asked the following question: “To what extent are you currently having each of these experiences?” They focus on their general perceptions, feelings or attitudes rather than their coping responses to a specific stressor. Items are rated on an 11-point Likert scale (0 = *not at all*; 11 = *extremely*). The scale was adapted to Polish conditions by Zarzycka. It includes four subscales (Zarzycka 2014). Religious comfort (PR) concerns the sense of trust toward God, perceiving God as almighty, supportive person and taking care of people, perceiving faith as a source of strength, harmony, peace and sense of meaning. Negative emotions toward God (NEB) include negative feelings toward God; perceiving God as unfair, cruel, untrustworthy, and abandoning people. Fear–guilt (LW) concerns preoccupation with one’s own guilt, sin; feeling unforgiven by God. Negative social interactions surrounding religion (NISR) includes negative emotions and relationships with fellow congregants. Cronbach's alpha coefficient for individual subscales it was: 0.59 ≤ α ≤ 0.96. In the current study, Cronbach's alpha coefficient is: 0.71 ≤ α ≤ 0.91.

## Results

### Theoretical Validity of the SAG

**Fig. 1:**
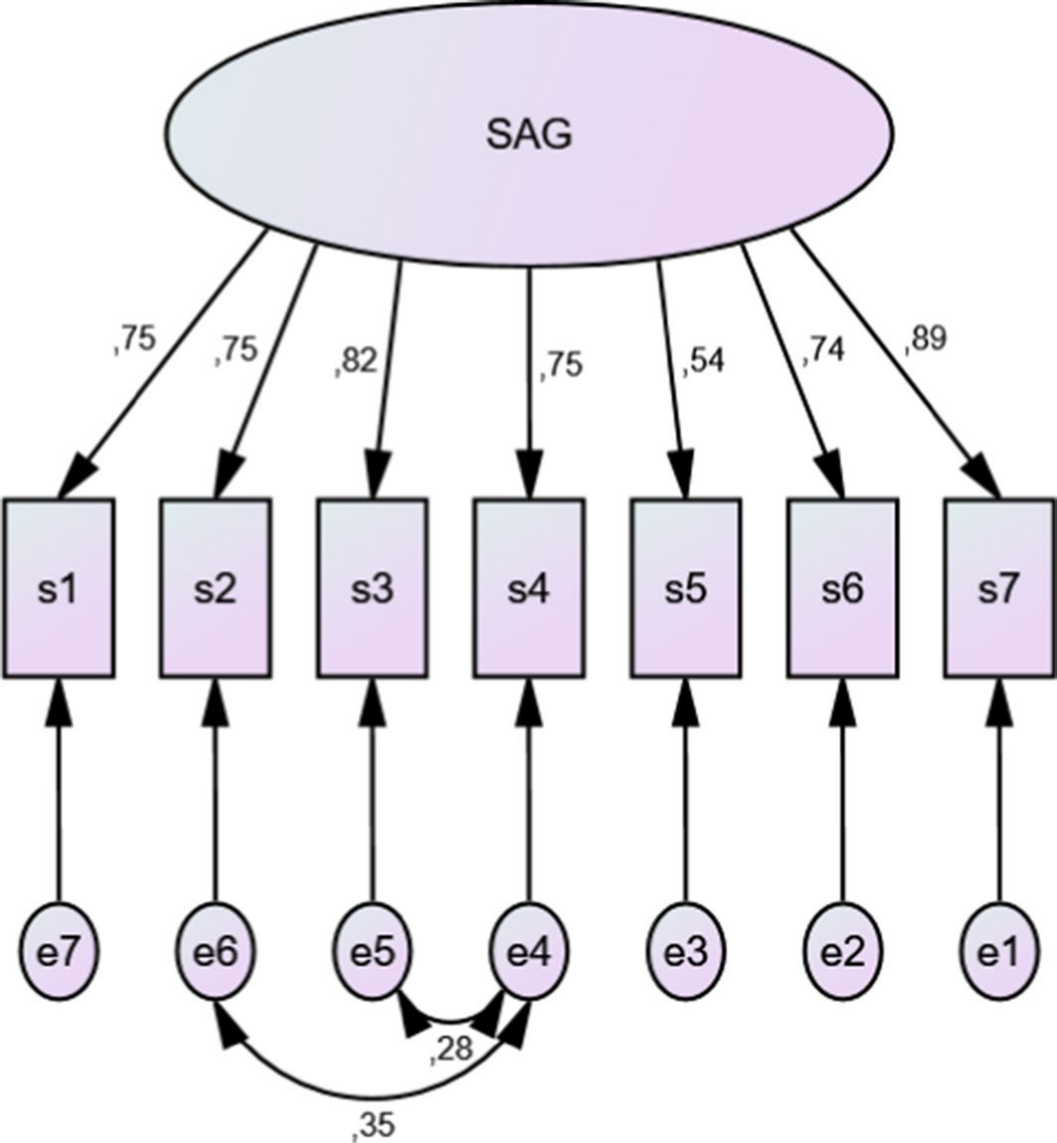
The factor confirmation analysis model for the SAG. At the arrows, the factor loadings of the scale items are placed (*N* = 200)

The values of the scale parameters of SAG were estimated and verified by the Maximum Likelihood (MLR) method applying confirmatory factor analysis of the SPSS Amos package. The hypothesis 2 was verified by one-factor solution, which reveals adequate fit. The properties of the confirmatory factor analysis are presented in Table 4, and the loadings of individual statements are presented in Fig. 1.

**Table 4:** Coefficients of goodness of fit in the model of confirmatory factor analysis of the SAG (*N* = 200)

One-factor model	CMIN/df	GFI	AGFI	CFI	TLI	RMSEA
	1.53	.97	.93	.99	.98	.05

CFI, Comparative fit index; AGFI, Adjusted goodness of fit index; GFI, Goodness of fit index; TLI, Tucker-Lewis Index; RMSEA, Root mean square error of approximation

The satisfactory fit is demonstrated by all the indices of goodness of fit considered in this study, including RMSEA = 0.05 (Marsh and Hocevar 1985). In addition, Cronbach's value (0.90) for scale is satisfactory, which shows a high reliability of the measure. Thus, it could be concluded that the validity of the scale has been confirmed. This indicates that the structure of the scale is well-defined and plausible. It also suggests that the current scale meets the required psychometric parameters.

### Criterion Validity of the SAG

According to the literature of the subject, as well as to earlier studies (Głaz 1998; Krok 2015; Zarzycka 2018), the religious experience of God's abandonment ought to have a significant statistical and positive relationship with such elements of Christian religiosity as interest in religious issues, religious beliefs, prayer, religious experience, worship, religious comfort, and negative relationship with fear–guilt, negative emotions toward God, and negative social interactions surrounding religion. The correlations obtained in the SAG and The Centrality of Religiosity Scale (CRS), and also Religious Comfort and Strain Scale (RCSS) confirmed the mentioned relations and therefore the criterion validity. Pearson's correlation coefficients *r* are presented in Table 5.

**Table 5:** Pearson's *r* correlation values received for the SAG, the CRS, and the RCSS (*N* = 200)

Variables	ZPR	PR	M	DR	K	RC	NEB	LW	NISR
SAG	.42**	.75**	.69**	.67**	.68**	.77***	.-37**	.-15*	.− 37**

SAG, Scale of Abandonment by God; ZPR, Interest in religious issues; PR, Religious beliefs; M, Prayer; DR, Religious experience; K, Worship; RC, Religious comfort; NEB, Negative emotion; LW, Fear-guilt; NISR, Negative social interactions

*p < .05; **p < .01; ***p < .001

Explanation of the results received in the SAG, in the CRS and in the RCSS, displays (Table 5) that a statistically significant positive relationship takes place between the SAG and interest in religious issues (ZPR) (*r* = 0.42, *p* < 0.01), religious beliefs (PR) (*r* = 0.75, *p* < 0.01), prayer (M) (*r* = 0.69, *p* < 0.01), religious experience (DR) (*r* = 0.67, *p* < 0.01), worship (K) (*r* = 0.68, *p* < 0.01), religious comfort (RC) (*r* = 0.77, *p* < 0.001). Moreover, a significant negative statistical relationship is recorded between the SAG and fear-guilt (LW) (*r* = − 0.15, *p* < 0.05), negative emotion (NEB) (*r* = − 0.38 *p* < 0.01), and negative social interactions (NISR) (*r* = − 0.37 *p* < 0.01).

## General Discussion

It must be said that the subjective feeling of being abandoned by God has positive impact on certain aspects of the life of contemporary man. The current article presents the problems of religiosity and spirituality of contemporary man, which can be measured by the self-assessed instrument, SAG as a simple, 7 items, short and psychometric validated scale. The SAG allows studying the Catholic religious experience of abandonment by God of people that live in Catholic religious surroundings.

According to the methodology, the development of SAG was conducted following the procedure: a) the extent to which the scale measures the adapted theoretical concept that is the experience of abandonment by God, and b) verification of the validity of the scale. First, positive statements related to the Catholic concept of an abandoned experience by God were generated and developed. Secondly, the exploratory factor analysis was used revealing one-factor solution (Table 1). Third, the theoretical validity of the scale was assessed by using confirmatory factor analysis (Fig. 1 and Table 4) and the criterion validity (Table 5).

The analysis shows that the scale has satisfactory structure.

- The scale has high reliability indices. The scale items explained 47,8% total variance of SAG in factor analysis solution. The confirmatory factor analysis confirmed a good fit of one-factor model (RMSEA = 0.05) and displays that the factor loadings of the items are satisfactory high. The range of loadings of confirmatory factor analysis for particular items is from 0.54 to 0.89 (Fig. 1) and is similar to the range of loadings received in the exploratory factor analysis (0.622–0.872; Table 1). It should be stated that the proposed SAG one-factor model of experience of abandonment by God is satisfactory.
- Criterion validity of the SAG was determined by the correlations with other adequate tools as The Centrality of Religiosity Scale (CRS) and Religious Comfort and Strain Scale (RCSS) (Study 3). As expected (Jaworski 1989; Głaz 1998, 2013b), the SAG correlates statistically significantly and positively with all dimensions of the CRS. The correlation coefficients of CRS dimensions of religiosity with the SAG range from 0.42 to 0.75. On the other hand, the SAG correlates negatively and significantly with three factors of the RCSS: negative emotion (NEB), negative social interactions (NISR), and fear-guilt (LW), and with one-factor positively: religious comfort (RC). The correlation coefficients of the RCSS with the SAG range from 0.15 to 0.77.

The analysis of the issue raised several remarks.

In the validation process, a 7-item instrument, SAG was created to measure the experience of God abandonment. Despite the strong evidence of the instrument's reliability and validity, cautions should be taken in generalizing the results because the samples in this study are representative to the Catholic population. In the future, the current structure of the scale may be enhanced by adding new items. In addition, the use of the SAG test in other cultural environments will require its cultural adaptation, according to the psychometric methodology.

The research reveals that contemporary man experiences God's abandonment, which can be measured empirically. The SAG differs from some measures of religiosity and spirituality because it measures the experience of the abandonment by God, which can have the influence on psychological well-being in the life of the believers and play also a positive role in their lives. The scale may be recommended for further use among Catholics of other nationalities and may provide valuable cross-cultural and inter-religious comparisons in the empirical psychology of religion.

## Conclusion

This study presents an exploratory and validation analysis to validate the proposed measuring instrument, the SAG scale and fully confirmed the stated hypotheses that experience of God's abandonment in the life of contemporary Catholic man is satisfactory measured by SAG, which has the sufficient psychometric properties. It means that the both hypotheses H_1_ and H_2_ are fully confirmed.

Despite the limitations, the evidence presented here shows that the SAG is a promising self-assessment instrument of the daily religiosity of Catholics, thanks to which counselors, psychologists and clergy/chaplains can better understand the religious problems of their followers and their relationship to any psychological outcomes like, well-being or clinical interests.

The included statements in the scale refer to the deep and mature religious life of an individual and religious groups with their consequences in personal and social life. The scale has a positive meaning and can be interpreted as a measure of the feeling of being abandoned by God, and as an encouragement to engage and develop certain aspects of human life.

The application of the current scale may have wide impact in the lives of believers. SAG can be used among young people and the elderly living in the environment of the Catholic religion. Due to its nature and purpose, it can be used to study the intensity of religious life, taking into account the positive feeling of being abandoned by God, and to determine to what extent it affects their individual and social life. It can be applied as a direct help in guiding religious development in high school and university students, as well as in the pastoral care of adults.

An important advantage of this method is that it concerns the experience of the abandonment by God within the Catholic religion and has a positive meaning. The high score in SAG suggests that a person, although accompanied by a feeling of being abandoned by God, believes that God is present in her or his life, trusts in God and strives to establish a relationship with God. She or he is accompanied by a feeling of longing for God, and are convinced that God loves man despite sins and neglects, and perceives her or his own life as valuable and meaningful.

The present study like many other scientific investigations has some limitations that must be considered when interpreting its results. The model fits one sample reported in the present investigation, which does not imply the ultimate solution.

Previous research and the present analysis of the issue indicate the multifaceted nature of Catholic religiosity and spirituality (Głaz 1998; Hill and Hood 1999). It is hoped that the results presented in the present study will stimulate researchers' interest in developing this new measure and can also be used to create another measure of Catholic religiosity and spirituality, taking into account other aspects, not yet analyzed.
